# A High-Power Broadband Terahertz Source Enabled by Three-Dimensional Light Confinement in a Plasmonic Nanocavity

**DOI:** 10.1038/s41598-017-04553-4

**Published:** 2017-06-23

**Authors:** Nezih Tolga Yardimci, Semih Cakmakyapan, Soroosh Hemmati, Mona Jarrahi

**Affiliations:** Electrical Engineering Department, University of California – Los Angeles, Los Angeles, CA 90095 United States

## Abstract

The scope and potential uses of time-domain terahertz imaging and spectroscopy are mainly limited by the low optical-to-terahertz conversion efficiency of photoconductive terahertz sources. State-of-the-art photoconductive sources utilize short-carrier-lifetime semiconductors to recombine carriers that cannot contribute to efficient terahertz generation and cause additional thermal dissipation. Here, we present a novel photoconductive terahertz source that offers a significantly higher efficiency compared with terahertz sources fabricated on short-carrier-lifetime substrates. The key innovative feature of this source is the tight three-dimensional confinement of the optical pump beam around the terahertz nanoantennas that are used as radiating elements. This is achieved by means of a nanocavity formed by plasmonic structures and a distributed Bragg reflector. Consequently, almost all of the photo-generated carriers can be routed to the terahertz nanoantennas within a sub-picosecond time-scale. This results in a very strong, ultrafast current that drives the nanoantennas to produce broadband terahertz radiation. We experimentally demonstrate that this terahertz source can generate 4 mW pulsed terahertz radiation under an optical pump power of 720 mW over the 0.1–4 THz frequency range. This is the highest reported power level for terahertz radiation from a photoconductive terahertz source, representing more than an order of magnitude of enhancement in the optical-to-terahertz conversion efficiency compared with state-of-the-art photoconductive terahertz sources fabricated on short-carrier-lifetime substrates.

## Introduction

Time-domain terahertz imaging and spectroscopy are of great interest to scientists and engineers because of their unique applications in chemical sensing, medical diagnosis, and industrial quality control^1–14^. However, the performance of these systems still cannot fully satisfy the needs of researchers. One of the main requirements for applications of these systems is the generation of high-power broadband terahertz pulses through a compact setting.

Among the various terahertz sources that are used to generate pulsed terahertz radiation, photoconductive terahertz sources are typically used in compact time-domain terahertz imaging and spectroscopy systems^15–20^. A conventional photoconductive source consists of a terahertz antenna fabricated on a photoconductive semiconductor substrate. When the device is pumped by a femtosecond optical pulse and a bias voltage is simultaneously applied via the antenna arms, the photo-generated carriers drift to the antenna contact electrodes. This generates an ultrafast current within the device. Pulsed terahertz radiation is generated as this current drives the antenna.

The main problem facing such photoconductive sources is the limited number of photo-generated carriers that can effectively contribute to the pulsed terahertz radiation. Even when a sufficient bias voltage is applied to provide a high electric field for drifting the carriers, only carriers generated within a few hundred nanometers of the contact electrodes can drift to the antenna within a sub-picosecond time-scale. The remaining carriers only add low-frequency components to the radiation and heat the source unnecessarily.

To prevent early thermal breakdowns, photoconductive substrates with carrier lifetimes of less than a picosecond are generally used^21–27^. This helps to recombine slow carriers before they are swept up by the contacts and consequently decreases the thermal dissipation within the device. However, techniques for growing short-carrier-lifetime semiconductor substrates rely on the addition of deep-level defects to the substrate lattice. This inherently reduces the carrier mobility and drift velocity and, hence, the optical-to-terahertz conversion efficiency of the terahertz source.

In this article, we present a novel photoconductive terahertz source that overcomes the slow carrier problem by means of a nanocavity that induces the three-dimensional confinement of the optical pump light around the terahertz radiating elements. The presented terahertz source is fabricated on a thin, low-defect, high-mobility semiconductor layer, which is grown on a substrate with a distributed Bragg reflector (DBR) structure. It consists of a two-dimensional array of plasmonic nanoantennas fabricated on the active high-mobility semiconductor layer. These plasmonic nanoantennas are specifically designed to excite surface plasmon waves at the optical pump wavelengths, while serving as radiating elements at terahertz frequencies^28^. The plasmonic structures and DBR layer are designed to form a nanocavity to tightly confine the optical pump beam inside the active high-mobility semiconductor layer. Therefore, when an optical beam pumps the device, almost all carriers are generated in very close proximity to the nanoantennas. If a bias voltage is simultaneously applied, almost all of the photo-generated carriers can reach the nanoantennas and contribute to generating an ultrafast current. As this strong, ultrafast current drives the terahertz nanoantennas, a much more efficient optical-to-terahertz conversion occurs compared to the state-of-the-art sources based on short-carrier-lifetime substrates, and a powerful terahertz pulse can be generated. In addition, by mitigating the effect of slow carriers, robust operation of this source can be achieved.

We compare this source with a similar plasmonic photoconductive nanoantenna array fabricated on a short-carrier-lifetime substrate. The presented nanocavity-enhanced plasmonic nanoantenna array produces a photocurrent that is 36 times stronger and, consequently, terahertz radiation that is 62 times stronger compared with those observed for the device based on the short-carrier-lifetime substrate. Using the presented photoconductive terahertz source, we achieve a record-high power of 4 mW for pulsed terahertz radiation over the 0.1–4 THz frequency range.

**Figure 1:**
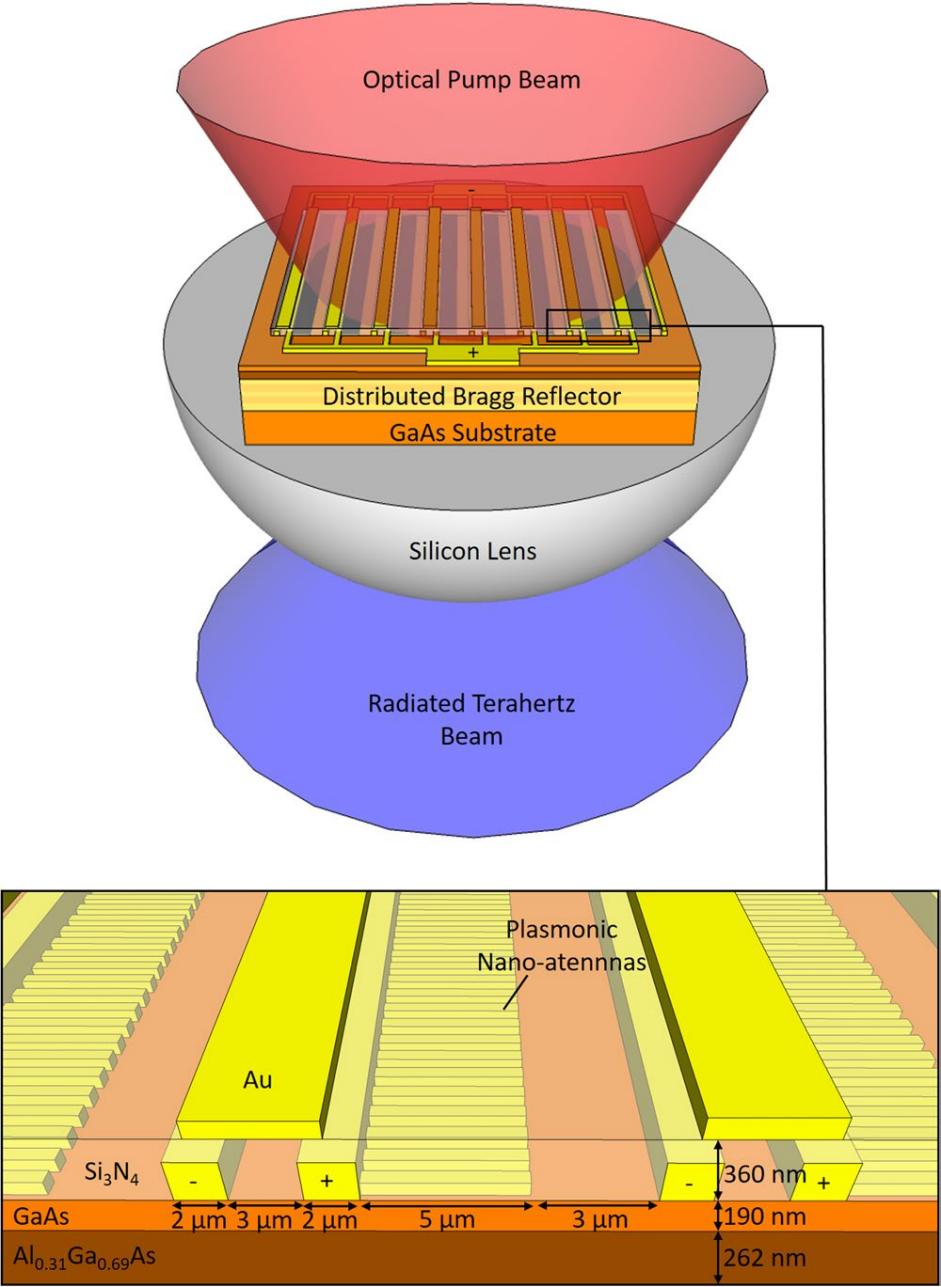
Schematic diagram and operation principle of the presented photoconductive source based on a nanocavity formed by a plasmonic nanoantenna array and DBR structure.

A schematic diagram of the presented terahertz source is illustrated in Fig. 1. The source is mounted on a hemispherical silicon lens with a diameter of 12 mm. The DBR structure is grown on a semi-insulating GaAs substrate. It consists of 25 alternating pairs of 60 nm thick AlAs and 55 nm thick Al_0.33_Ga_0.67_As layers. The plasmonic nanoantenna array is fabricated over an area of 1 × 1 mm^2^ on the undoped top GaAs layer of 190 nm in thickness. A 262 nm thick Al_0.31_Ga_0.69_As layer is used as a buffer layer between the top active GaAs layer and the DBR. Each plasmonic nanoantenna array is connected to the anode bias lines to take advantage of high-speed electrons rather than holes. A silicon nitride anti-reflection coating is deposited on the nanoantennas to further enhance the transmission of optical pump beam to the top active GaAs layer. Every other gap between the anode and cathode bias lines is shadowed by an additional layer of metal deposited on top of the anti-reflection coating. This prevents generation of any ultrafast current in the opposite direction to the current driving the nanoantennas.

**Figure 2:**
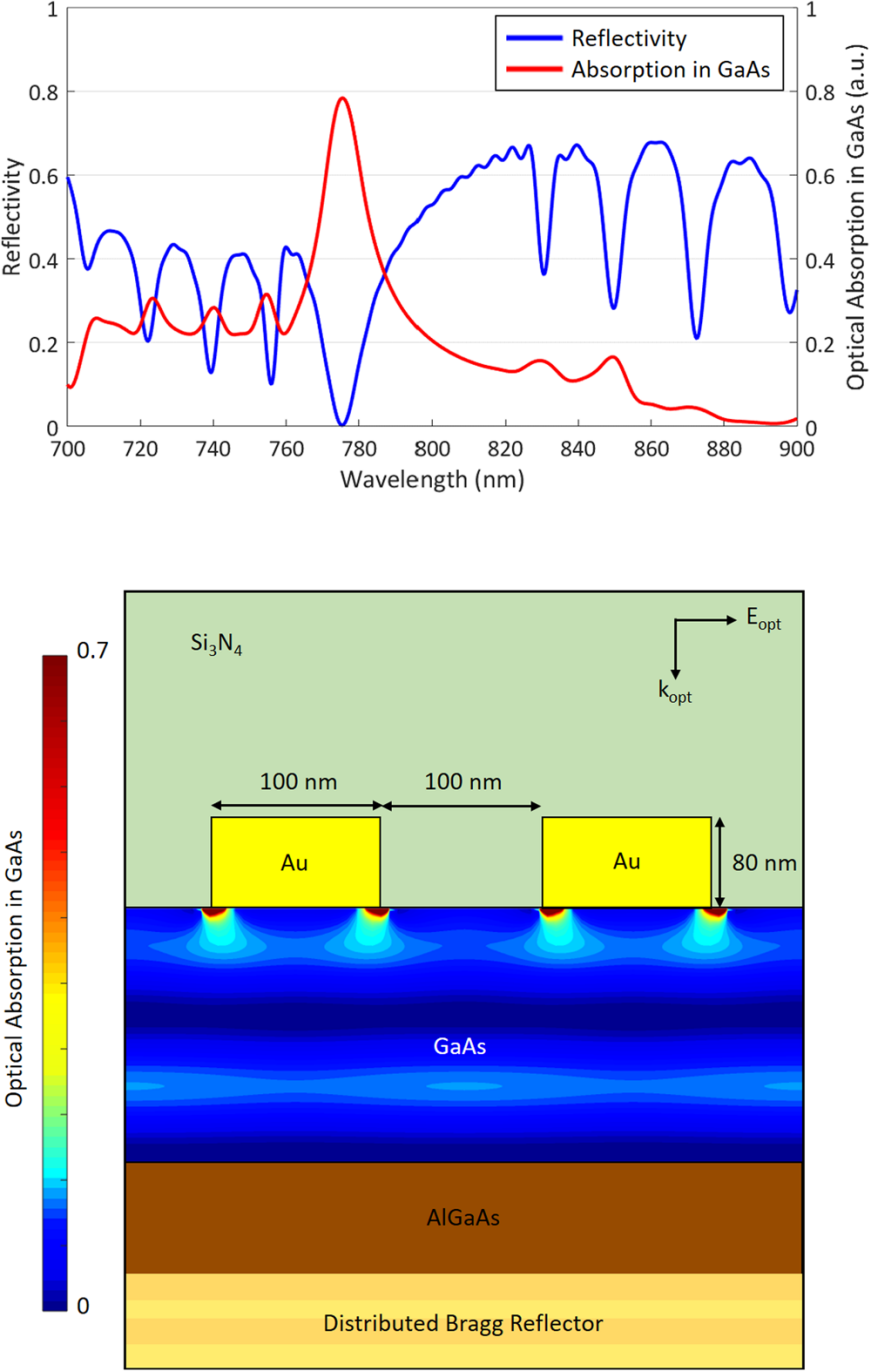
(**a**) The reflectivity of the designed plasmonic nanoantennas and the optical absorption in the top GaAs region as a function of the wavelength. (**b**) Two-dimensional color plot of the optical absorption in the top GaAs region at a wavelength of 775 nm.

**Figure 3:**
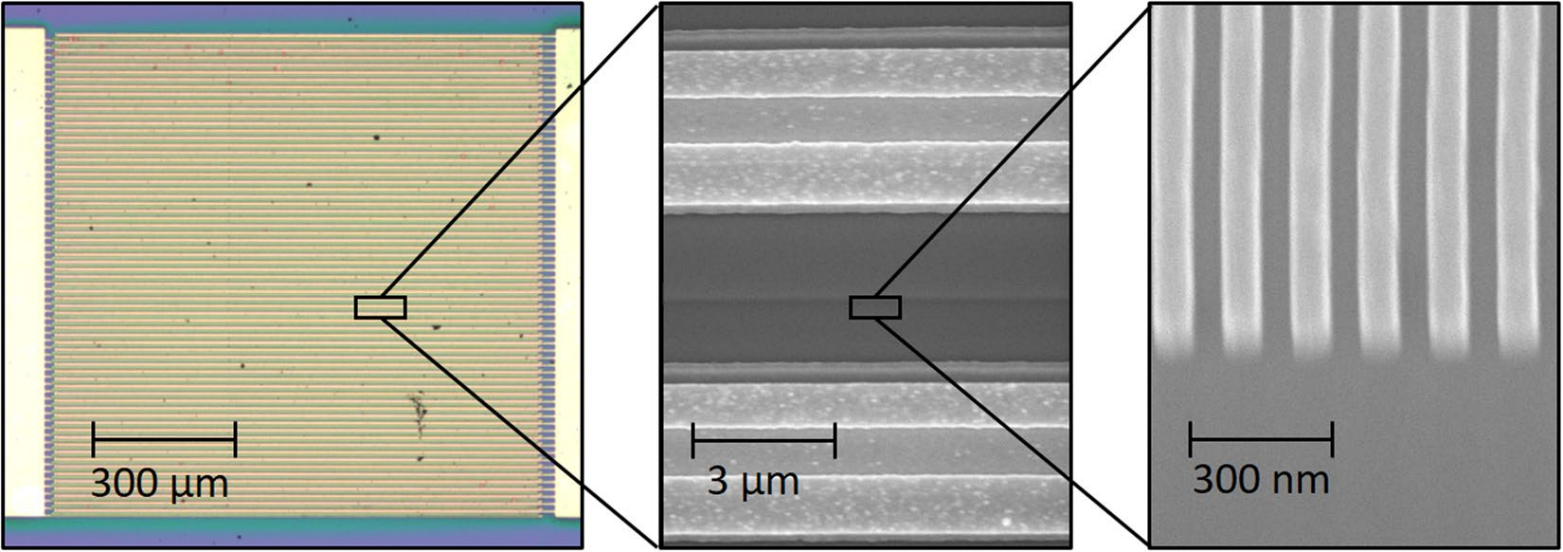
Optical microscopy image of the terahertz source prototype and scanning electron microscopy images of the bias lines, shadow metals and plasmonic nanoantennas.

The plasmonic nanoantennas and DBR structure are designed to confine the incident optical pump beam in the top active GaAs layer. The interaction between plasmonic nanoantennas integrated with this particular semiconductor structure and a TM-polarized optical pump beam is analyzed using an electromagnetic simulation software based on the finite-difference time-domain method (Lumerical). The analysis is performed for a grating-shaped Au plasmonic nanoantenna array with a periodicity of 200 nm, a metal width of 100 nm and a metal height of 80 nm as well as a 360 nm thick Si_3_N_4_ anti-reflective coating. The grating geometry is specifically selected to achieve zero optical reflection at a pump wavelength of 775 nm (Fig. 2a). This means that all incident photons at this wavelength are trapped inside the semiconductor. Of the 775 nm incident photons, 78% are absorbed by the top active GaAs layer, whereas the rest are dissipated as a result of plasmonic losses. A two-dimensional color plot of the optical absorption inside the substrate at this wavelength is shown in Fig. 2b. The enhancement in the optical absorption immediately below each nanoantenna proves that optical transmission is accompanied by the excitation of surface plasmon waves at 775 nm. The formation of a standing wave in the substrate indicates the resonant nature of the designed nanocavity. Considering that the entirety of the optical absorption occurs only in the top 190 nm thick GaAs volume immediately below the nanoantennas, most carriers will contribute to the radiation at terahertz frequencies, and there will be almost no carriers adding low-frequency components to the radiation in such a device structure. To achieve broadband radiation from the terahertz nanoantennas, the length of the nanoantennas is chosen to be much smaller than the wavelengths of the terahertz spectral region (5 μm). Figure 3 shows an optical microscopy image of a fabricated terahertz source and scanning electron microscopy images of its plasmonic nanoantennas. The device fabrication process is described in the methods section.

## Results

**Figure 4:**
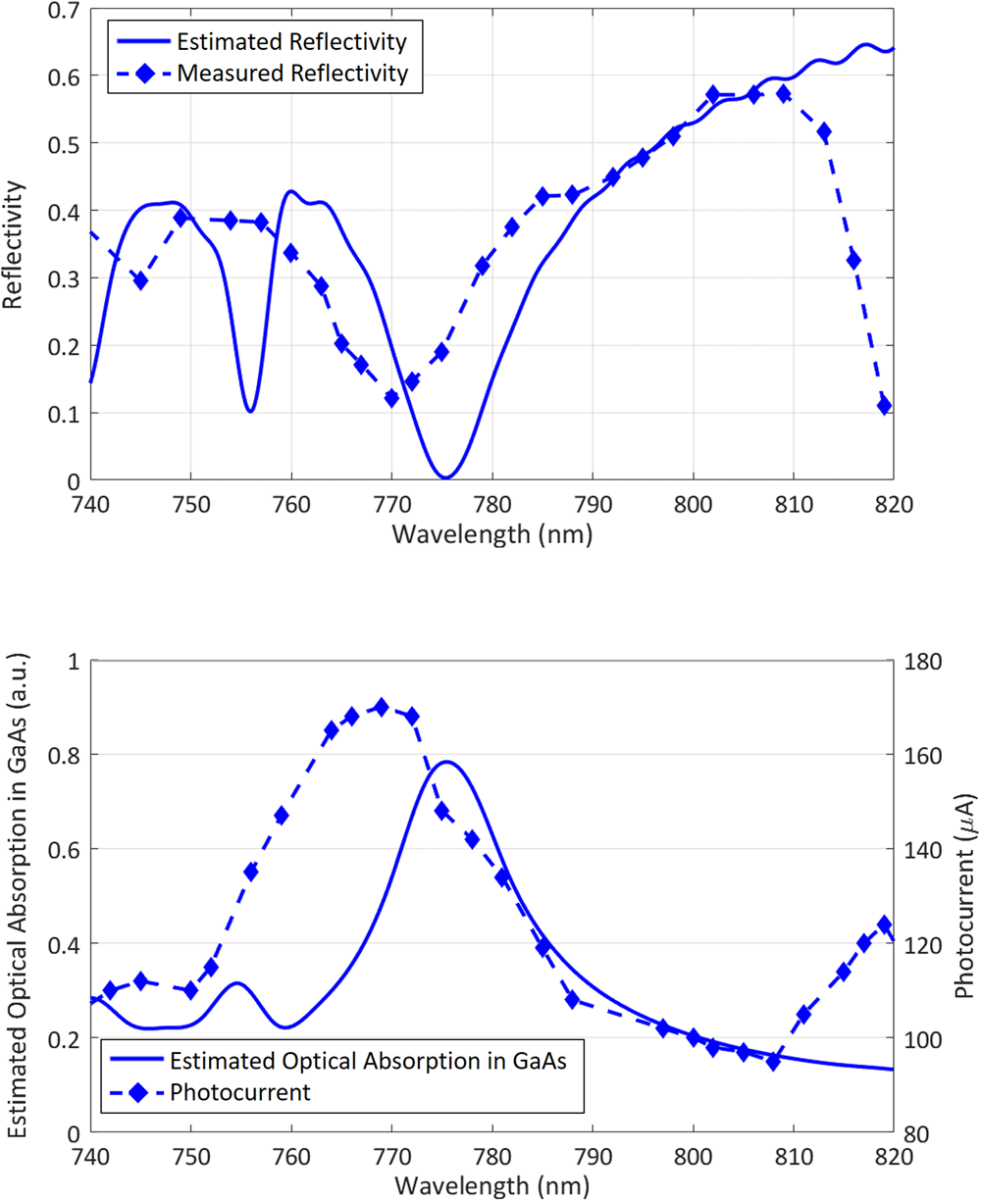
(**a**) The estimated and measured optical reflectivities of the plasmonic nanoantennas as a function of the wavelength. (**b**) The estimated optical absorption in the top GaAs layer and the measured photocurrent in the terahertz source prototype.

To experimentally prove that the nanocavity operates as designed, the optical reflection from the plasmonic nanoantennas is measured. To measure the optical reflectivity, another sample consisting only of plasmonic nanoantennas with a length of 0.1 mm and a Si_3_N_4_ anti-reflection coating, covering an area of 0.1 × 0.1 mm^2^, is fabricated on the same substrate. A Ti:sapphire laser is used in continuous-wave (CW) operation mode. The polarization of the optical beam is set normal to the nanoantennas, and the spot size of the beam is adjusted to focus on the area covered by the plasmonic nanoantennas. The reflectivity is measured under an optical pump power of 10 mW, and the wavelength of the incident pump beam is swept from 740 nm to 820 nm. The measured and estimated optical reflectivity from the simulation results are compared in Fig. 4a. The minimum measured reflectivity (~10%) is observed at 770 nm, corresponding to a slight blue shift compared with the estimated reflectivity. The increase in the minimum reflectivity and the shift in wavelength can be attributed to fabrication imperfections and small deviations in the thickness and composition of the grown semiconductor substrate layers from the intended design.

Subsequently, the photocurrent in the fabricated terahertz source prototype is measured using a CW optical beam with the same polarization. An optical pump power of 10 mW is used to illuminate the device. A bias voltage of 5 V is applied via the bias lines. As expected, a shift in wavelength similar to that observed for the reflectivity is also seen between the maximum measured photocurrent and the maximum optical absorption in the top GaAs layer as estimated from the simulations, for the same reasons described above (Fig. 4b).

To demonstrate the superior performance of the presented terahertz source, a plasmonic terahertz nanoantenna array is fabricated on a low-temperature-grown GaAs (LT-GaAs) substrate with a carrier lifetime of 0.3 ps^26^. The same device area, the same ratio between the active and shadowed areas, and the same nanoantenna length are chosen for this device to ensure a fair comparison.

To measure the terahertz radiation characteristics, the operation mode of the Ti:sapphire laser is changed to mode-locked and the fabricated terahertz sources are pumped by 135 fs wide optical pulses with a 76 MHz repetition rate, at a center wavelength of 770 nm.

**Figure 5:**
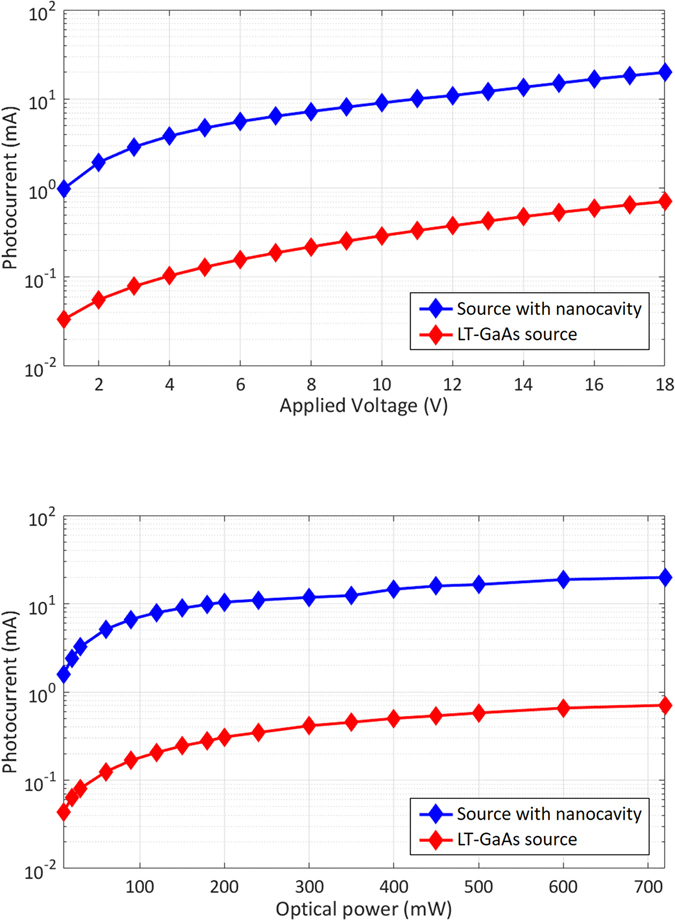
(**a**) Photocurrents in the nanocavity-based terahertz source and the terahertz source fabricated on the LT-GaAs substrate as a function of the bias voltage under an optical pump power of 720 mW. (**b**) Photocurrents in the nanocavity-based terahertz source and the terahertz source fabricated on the LT-GaAs substrate as a function of the optical pump power under a bias voltage of 18 V.

First, the photocurrents in the terahertz sources are measured. Figure 5a shows the measured photocurrent as a function of the bias voltage under an optical pump power of 720 mW. The nanocavity-based source exhibits a photocurrent that is 28 times stronger than that of the source fabricated on the LT-GaAs substrate. This stronger photocurrent can be attributed to the tighter light confinement near the plasmonic nanoantenna arrays and the higher carrier drift velocity offered by the low-defect semiconductor used in the nanocavity-based source. Figure 5b shows the photocurrent as a function of the optical pump power under a bias voltage of 18 V. At an optical pump power of 10 mW, the photocurrent is enhanced by a factor of 36 for the nanocavity-based source compared with the source fabricated on the LT-GaAs substrate. The enhancement factor slightly decreases to 28 under an optical pump power of 720 mW. This slight reduction can be attributed to the stronger carrier screening and scattering due to the larger number of photo-generated carriers in the active region of the nanocavity-based source compared with the carriers in the LT-GaAs-based source.

**Figure 6:**
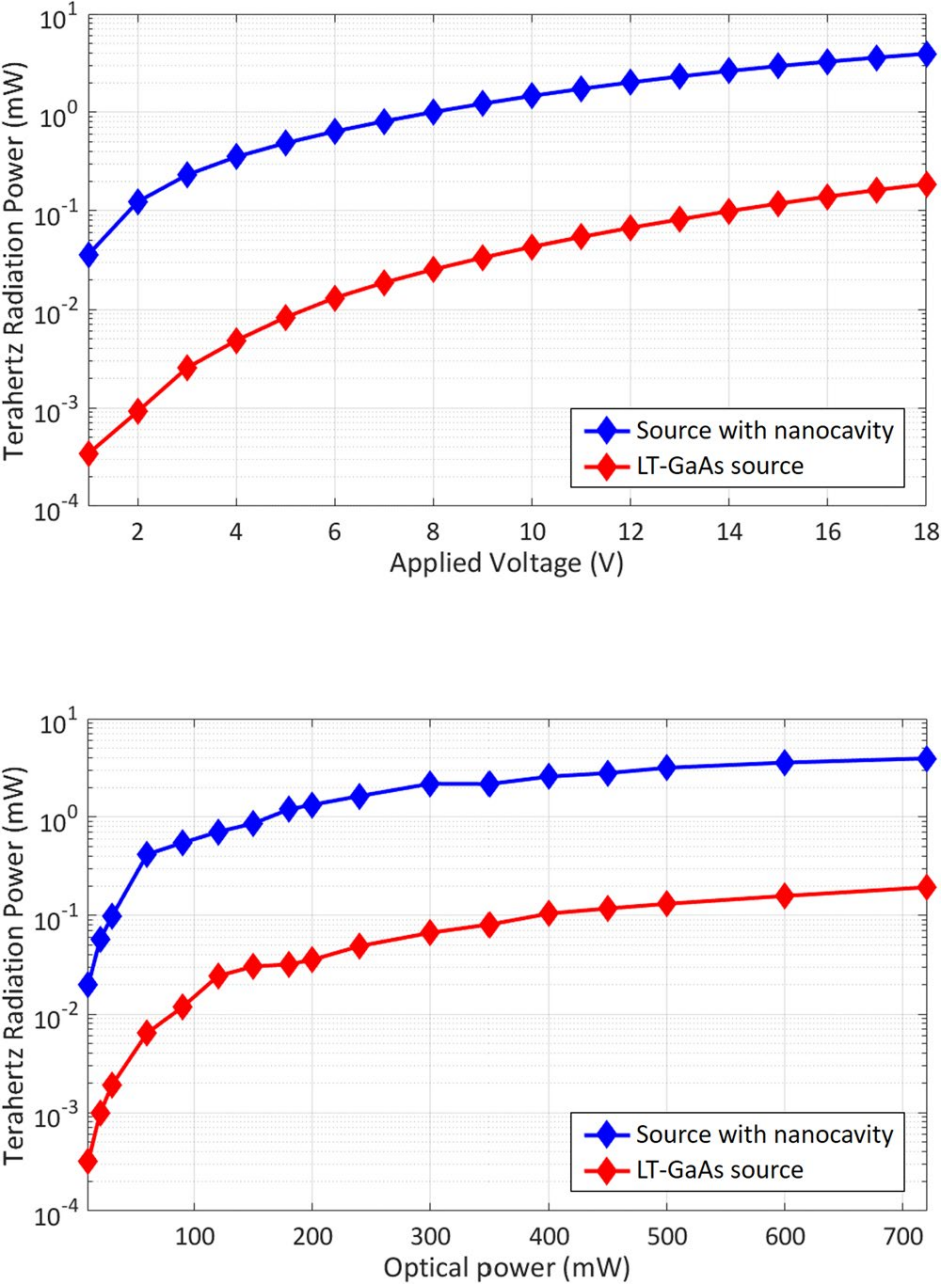
(**a**) Radiated terahertz power levels of the nanocavity-based terahertz source and the terahertz source fabricated on the LT-GaAs substrate as a function of the bias voltage under an optical pump power of 720 mW. (**b**) Radiated terahertz power levels of the nanocavity-based terahertz source and the terahertz source fabricated on the LT-GaAs substrate as a function of the optical pump power under a bias voltage of 18 V.

The radiated terahertz power is measured using pyroelectric detectors. Power levels higher than 20 μW are measured using a pyroelectric detector from Applied Physics and Electronics, Inc. (A.P.E. THz-30) calibrated at the Physikalisch-Technische Bundesanstalt (the German National Metrology Institute). Power levels lower than 20 μW are measured using another pyroelectric detector from Spectrum Detector, Inc. (SPIA-65 THz) calibrated using the A.P.E. THz-30 pyroelectric detector at a terahertz radiation power level of 20 μW. Figure 6a shows the radiated terahertz power levels of the two sources as a function of the bias voltage under an optical pump power of 720 mW. The measured terahertz radiation from the nanocavity-based source is 21 times stronger than that from the source fabricated on the LT-GaAs substrate. Figure 6b shows the radiated terahertz power levels of the terahertz sources as a function of the optical pump power level under a bias voltage of 18 V. Higher enhancements in the optical-to-terahertz conversion efficiency are observed at lower optical pump powers compared with those seen under stronger illumination. In fact, under an optical pump power of 10 mW, the nanocavity-based source produces terahertz radiation with an optical-to-terahertz conversion efficiency that is 62 times higher than that of the source fabricated on the LT-GaAs substrate, whereas this ratio decreases to 21 at an optical pump power of 720 mW. This reduction can again be attributed to the carrier screening and scattering induced by the high carrier concentration in the nanocavity at high optical pump power levels. These effects could be reduced by fabricating a larger area device and using an optical pump beam with a larger spot size.

**Figure 7:**
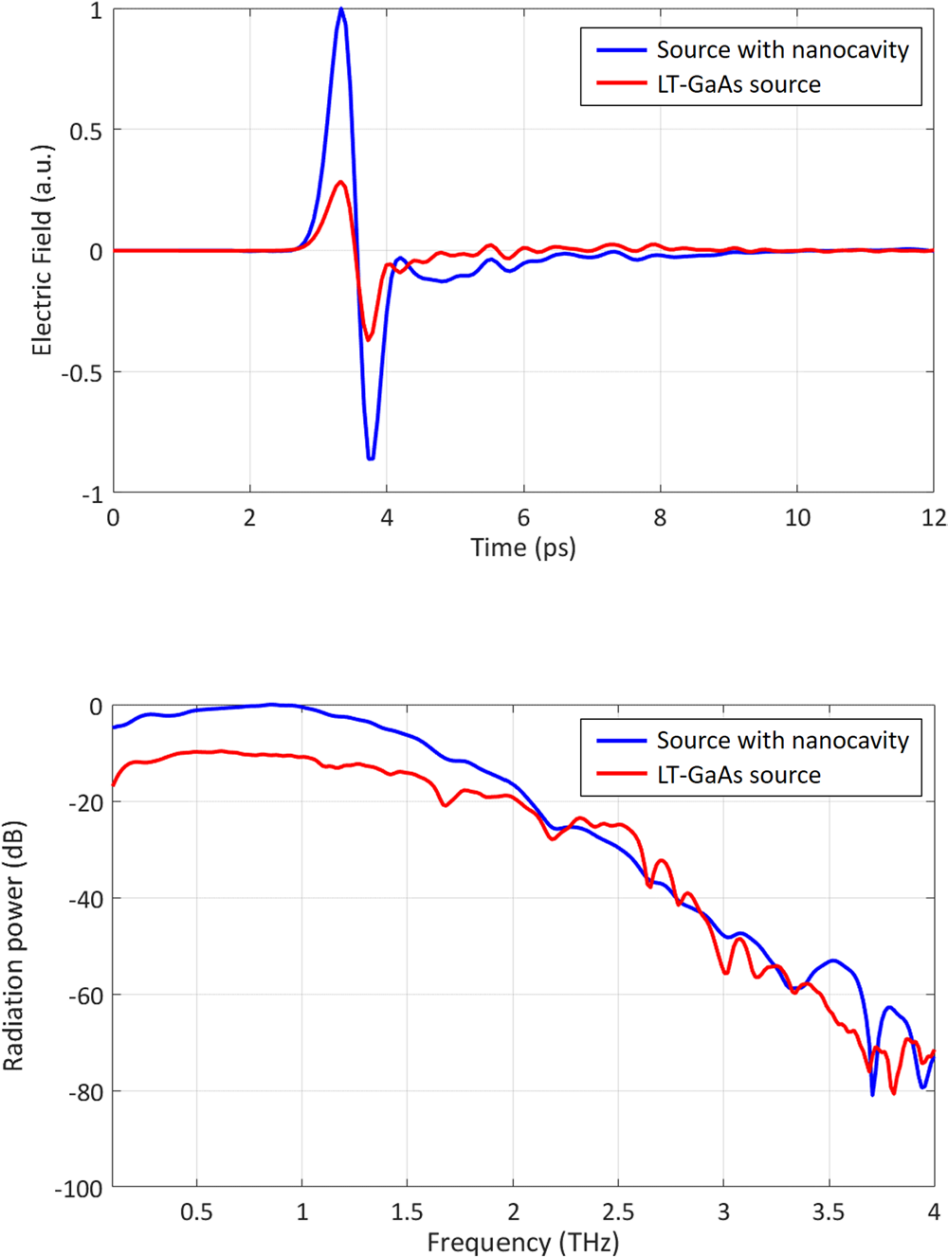
(**a**) The radiated electric fields and (**b**) the corresponding radiated power spectra of the nanocavity-based terahertz source and the terahertz source fabricated on the LT-GaAs substrate.

The radiation fields from the terahertz sources are characterized using a time-domain terahertz spectroscopy system. The electro-optic detection method is applied using a 0.5 mm thick ZnTe crystal. An optical pump power of 720 mW and a bias voltage of 18 V are applied to both terahertz sources. Terahertz radiation is focused onto the electro-optic crystal using polyethylene terahertz lenses. Figure 7a shows the measured radiation fields from both sources. Pulses with a full width at half maximum of 0.45 ps are observed from both devices. The nanocavity-based terahertz source offers a peak-to-peak field that is 3.7 times stronger than that of the terahertz source fabricated on the LT-GaAs substrate. The radiation power spectra are calculated by taking the fast Fourier transforms of the radiation fields (Fig. 7b). The nanocavity-based terahertz source offers 10 dB higher terahertz radiation power levels than the source fabricated on the LT-GaAs substrate for frequencies below 2 THz. This can again be attributed to the tighter light confinement near the plasmonic nanoantenna arrays and the higher carrier drift velocity offered by the low-defect semiconductor used in the nanocavity-based source. Similar radiation power levels are observed at frequencies above 2 THz. Only carriers generated within a few tens of nanometers distance from the nanoantennas can contribute to these high frequencies, and the carriers in this region are mostly generated via optical transmission by means of surface plasmon waves. Since the plasmonic nanoantennas in both sources offer similar levels of optical absorption enhancement in this region, the radiation power levels at frequencies above 2 THz are expected to be similar. Radiation is observed throughout a broad frequency range extending up to 4 THz from both sources.

In summary, we present a novel photoconductive terahertz source that offers record-high-power terahertz pulses with significantly higher optical-to-terahertz conversion efficiencies compared to the state-of-the-art. The key innovative feature of the presented terahertz source is the strong confinement of the optical pump beam to a very small volume immediately below the terahertz radiating elements. This confinement is achieved by means of a nanocavity formed by a DBR and plasmonic nanoantenna array. This structure is beneficial for eliminating the slow carriers found in conventional photoconductive sources since almost all carriers are generated in very close proximity to the nanoantennas and effectively contribute to the pulsed radiation at terahertz frequencies. Another advantage of this structure is that it eliminates the need to use a low-mobility, short-carrier-lifetime semiconductor substrate as the active photoconductor. By enabling a much more efficient light confinement near the terahertz radiating elements and benefiting from higher carrier drift velocities offered by low-defect semiconductors, the presented nanocavity-based photoconductive terahertz source generates much stronger terahertz radiation compared with the state-of-the-art sources fabricated on short-carrier-lifetime semiconductor substrates. Using the nanocavity-based photoconductive terahertz source, a record-high power of 4 mW for pulsed terahertz radiation is achieved at an optical pump power of 720 mW over the 0.1–4 THz frequency range. This represents more than an order of magnitude of enhancement in the optical-to-terahertz conversion efficiency compared with state-of-the-art photoconductive terahertz sources fabricated on short-carrier-lifetime substrates. We believe that such a high-power broadband terahertz source can play a key role in future applications of time-domain terahertz spectroscopy.

## Methods

### Terahertz source fabrication process

The DBR structure, the AlGaAs buffer layer and the top GaAs layer are grown on a GaAs substrate via molecular beam epitaxy and used for fabricating a terahertz source prototype. The fabrication process begins with electron beam lithography for patterning the plasmonic terahertz nanoantennas. A metal evaporator is used to deposit a 2 nm thick Ti adhesive layer and a 78 nm thick Au layer. This is followed by a lift-off process for the formation of the nanoantennas. Subsequently, optical lithography is used to pattern the anode and cathode bias lines. Then, 50 nm/550 nm Ti/Au bias lines are formed by depositing the metals via evaporation, followed by a lift-off process. A 360 nm thick Si3N4 anti-reflection layer is coated onto the device via plasma-enhanced chemical vapor deposition. Then, 10 nm/90 nm Ti/Au shadow metals are patterned via optical lithography, deposited via evaporation and formed using a lift-off process. The contact vias are opened by etching the Si3N4 layer via reactive-ion etching.
